# An adverse outcome pathway for immune-mediated and allergic hepatitis: a case study with the NSAID diclofenac

**DOI:** 10.1007/s00204-020-02767-6

**Published:** 2020-05-05

**Authors:** Saravanakumar Selvaraj, Jung-Hwa Oh, Jürgen Borlak

**Affiliations:** 1 Centre for Pharmacology and Toxicology, Hannover Medical School, 30625 Hannover, Germany; 2 Department of Predictive Toxicology, Korea Institute of Toxicology, Gajeong-ro, Yuseong, Daejeon, 34114 Republic of Korea

**Keywords:** Adverse outcome pathways (AOP), Drug-induced liver injury (DILI), Diclofenac, Hepatotoxicity, Hepatitis, Immune-mediated, Inflammation, Immunoallergic

## Abstract

Many drugs have the potential to cause drug-induced liver injury (DILI); however, underlying mechanisms are diverse. The concept of adverse outcome pathways (AOPs) has become instrumental for risk assessment of drug class effects. We report AOPs specific for immune-mediated and drug hypersensitivity/allergic hepatitis by considering genomic, histo- and clinical pathology data of mice and dogs treated with diclofenac. The findings are relevant for other NSAIDs and drugs undergoing iminoquinone and quinone reactive metabolite formation. We define reactive metabolites catalyzed by CYP monooxygenase and myeloperoxidases of neutrophils and Kupffer cells as well as acyl glucuronides produced by uridine diphosphoglucuronosyl transferase as molecular initiating events (MIE). The reactive metabolites bind to proteins and act as neo-antigen and involve antigen-presenting cells to elicit B- and T-cell responses. Given the diverse immune systems between mice and dogs, six different key events (KEs) at the cellular and up to four KEs at the organ level are defined with mechanistic plausibility for the onset and progression of liver inflammation. With mice, cellular stress response, interferon gamma-, adipocytokine- and chemokine signaling provided a rationale for the AOP of immune-mediated hepatitis. With dogs, an erroneous programming of the innate and adaptive immune response resulted in mast cell activation; their infiltration into liver parenchyma and the shift to M2-polarized Kupffer cells signify allergic hepatitis and the occurrence of granulomas of the liver. Taken together, diclofenac induces divergent immune responses among two important preclinical animal species, and the injury pattern seen among clinical cases confirms the relevance of the developed AOP for immune-mediated hepatitis.

**Electronic supplementary material:**

The online version of this article (10.1007/s00204-020-02767-6) contains supplementary material, which is available to authorized users.

## Introduction

The liver is a primary target organ of toxicity, and drug-induced liver injury (DILI) is a major concern that results in box warnings and even withdrawals of drugs from the market. It is a leading cause for acute liver failure (Przybylak and Cronin 2012). Importantly, with the advent of omics platforms and high-throughput technologies a plethora of diverse data have become available to significantly advance the field of toxicological sciences. Given that “omics and NGS” platform technologies enable the capturing of whole genome/proteome information, the findings permit the construction of circuitries within a cell, tissue and organ in response to specific treatments across different species. Yet, turning data into knowledge remains a fundamental challenge.

The AOP concept represents a paradigm shift in regulatory toxicology and risk assessment (Vinken 2013); it is a conceptual framework that provides information concerning the causal relationship between an MIE and adverse outcome (AO) at different levels of biology, i.e., molecular, cellular/organelle, organ and whole organism (Ankley et al. 2010; Vinken et al. 2017). The construction of an AOP is based on mechanistic consideration and requires expert knowledge across different fields of biomedical science, i.e., molecular and cellular, development, frank organ toxicity/pathology and so forth.

AOPs also support the development of alternative testing strategies by endorsing the 3R principle in experimental works while the need for AOP network analysis arises from the complex biological processes underlying toxicological events. Ideally key event relationships across different species are defined with the weight of evidence being assessed by considering biological and mechanistic information. The AOP knowledgebase (AOP-KB, https://aopkb.oecd.org/) facilitates the search for mutual KEs and key event relationship (KER) components (Knapen et al. 2018; Pollesch et al. 2019), and to reflect the more complex processes in a toxicological insult AOP network analysis has been advocated. Such networks can be analyzed by linking the shared KE and KER components as a modular unit of the AOP (Knapen et al. 2018; Pollesch et al. 2019). Therefore, the sharing of modular units like KE and KER of individual AOPs is encouraged (Knapen et al. 2018), and these de facto AOP networks can cover lacking information. However, to explore the connectivity of shared modular units, the biological and mechanistic information needs to be considered in the context of taxonomy, life stage, sex and target organs.

In an effort to define an AOP for immune-mediated and allergic hepatitis, we queried the AOP knowledgebase for drug-induced hepatotoxicity. This revealed 8 AOPs linked to liver fibrosis, cholestasis and steatosis, and the associated MIE are defined by protein alkylation, LXR and inhibition of the bile salt export pump ABCB11. However, none of the AOPs are specific for immune-mediated and/or allergic hepatitis and the lack of mechanistic and clinical consideration poorly reflects the complexity of DILI. Notwithstanding, AOPs are living documents and will improve over time as updated information is effectively integrated to avoid oversimplifications. Moreover, continuous research in the underlying mechanism of DILI provides new insights which enable the construction of more detailed AOPs (Vinken 2015).

We report an AOP for immune-mediated and allergic hepatitis based on comprehensive data obtained from mouse and dog diclofenac repeated-dose studies and literature findings with clinical relevance. This non-steroidal anti-inflammatory drug (NSAID) exerts anti-inflammatory, analgesic and anti-pyretic effects through various mechanisms; however, its use can lead to adverse drug reactions including DILI (Banks et al. 1995). We entrained the AOP on our previous genomic studies as well as serum biochemistry, histopathology and immunohistochemistry and Western immunoblotting data (Lee et al. 2016; Selvaraj et al. 2017) and show that diclofenac reactive metabolism causes divergent immune responses among the two animal species commonly used in toxicity studies. We define iminoquinone and quinone reactive metabolites as MIE and confirm the relevance of these structural alerts for a larger group of drugs and chemicals undergoing iminoquinone and quinone reactive metabolite formation. Collectively, an AOP for hepatitis based on experimental and computational biology studies is presented.

## Methods

### Construction of AOP framework

Previously reported genomic data of diclofenac-induced liver injury in mice and dog models were interrogated to construct this AOP framework (male C57BL/6 mice with daily intraperitoneal injection of 30 mg/kg/day and 150 mg/kg/days for 14 days; male beagle dogs with daily oral dosing of 1 mg/kg/day and 3 mg/kg/day for 28 days) (Lee et al. 2016; Selvaraj et al. 2017). Given the complex inference resulting from on-target but exaggerated pharmacological responses and toxicity related to the physicochemical characteristics of diclofenac and its effects on cells, organelles, membranes and/or metabolic pathways, a combined approach was taken to define MIE. The subsequently performed computational analysis enabled the development of an AOP of immune-mediated hepatitis, and the concept is based on mechanistic plausibility. It recapitulates the pleiotropic effects induced by diclofenac treatment. Next to whole genome gene expression data, the weight of evidence includes histopathology, clinical chemistry and immunohistochemistry findings. Collectively, the strength, consistency and specificity of the AOPs are considered.

## Results and discussions

### Pharmacological mode of action of diclofenac

**Fig. 1:**
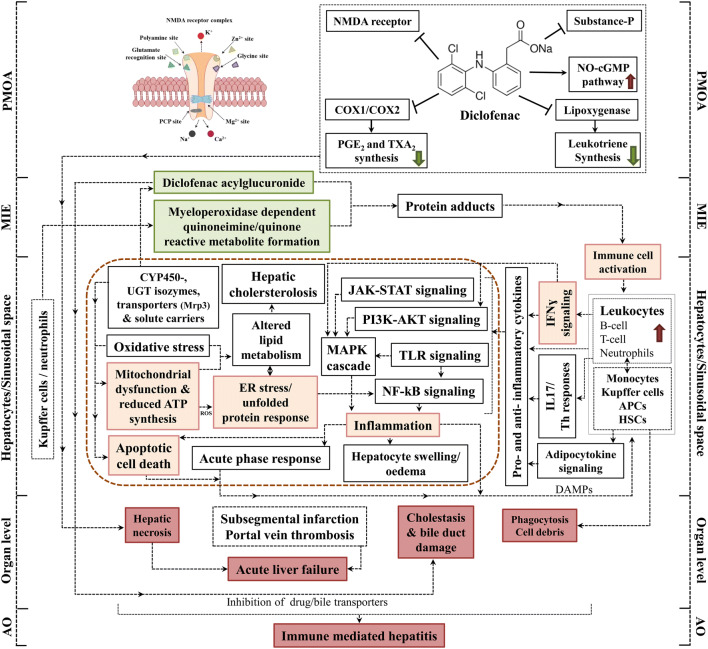
AOP of immune-mediated hepatitis in mice in response to diclofenac treatment. Diclofenac induced liver damage by the activation of complex immune-mediated inflammatory signaling cascades. This AOP illustrates the pharmacological mechanism of action of diclofenac and its adverse effects in liver. The MIE and KEs associated with immune-mediated hepatitis in mice are highlighted; the green boxes represent MIE, orange colored are KEs at the cellular level, and red-colored boxes define the KEs at the organ and organism level. The potential linkages among the KEs are based on experimental evidence and literature findings

Figure 1 illustrates the pharmacological mode of action of diclofenac. It inhibits cyclooxygenase 1 and 2 which catalyze arachidonic acid metabolism (Gan 2010). Likewise, it suppresses the production of leukotrienes by inhibiting lipoxygenases and prostaglandin E2 and thromboxane A2 synthesis (Gan 2010). Its analgesic activity resides in an activation of the nitric oxide–cGMP nociceptive pathway as well as inhibition of  N-methyl-d-aspartate (NMDA) receptor. Note, NMDA is a receptor for the neurotransmitter glutamate and diclofenac dampens NMDA-evoked nociceptor activity by modulating voltage-gated neuronal potassium channel activity. Likewise, activated NMDA receptors stimulate neuronal NO-synthase activity with increased production of NO. At the postsynaptic neuron NO activates the guanyl cyclase, and through retrograde diffusion NO reinforces the glutamatergic signaling in the presynaptic neuron thereby reinforcing nociception. NO may also induce an antinociceptive effect (Gan 2010). In addition, diclofenac suppresses activity of the neuropeptide substance P and is also a partial agonist of PPARɣ to augment lipogenesis (Gan 2010).

### AOP for immune-mediated hepatitis in mice

Diclofenac treatments caused an increase in serum biochemistry markers for liver injury (ALT and AST), and histopathology showed hepatomegaly, hepatocyte swelling, glycogen depletion, eosinophilic hepatocytes and cholesterolosis (Lee et al. 2016). Moreover, fatal acute liver failure was observed at a dose of 150 mg/kg. The genomic and biochemical validation study revealed immune-mediated responses to be considerably induced after diclofenac treatment (Lee et al. 2016).

#### MIE

Drug-induced hepatitis is a multistep process and frequently involves reactive metabolites which subsequently form drug–protein adducts. These are sensed by antigen-presenting cells to elicit innate and adaptive immune responses (Adams et al. 2010). Diclofenac is extensively metabolized by CYP monooxygenases and myeloperoxidases (MPO) of neutrophils and Kupffer cells into reactive metabolites notably quinoneimine intermediates (Table 1). The metabolism of diclofenac to reactive metabolites causes organ toxicity and this defines the MIE. A further example for a benzoquinone imine reactive metabolite causing liver injury is the metabolism of paracetamol to NAPQI. Correspondingly, NAPQI is the initial cause of hepatotoxicity and once again defines the MIE. Indeed, for a wide range of drugs quinoneimines are implicated in liver injury as summarized in supplementary Table S1.

**Table 1:** Summary of essential MIEs and KEs of immune-mediated hepatitis and hypersensitivity reactions in response to diclofenac

MIE/KEs	Evidence	Support for essentiality	References
*MIE*			
Metabolism, reactive metabolites including benzoquinone imine and acylglucuronide metabolites	High	• Diclofenac is metabolized to 3′-hydroxy-, 4′-hydroxy-, 5-hydroxy- 4′,5-dihydroxy-, and N,5-dihydroxydiclofenac by CYP2C9 and CYP3A4 and by the combined activity of CYP2C8 and UGT2B7 to yield acylglucuronides • Reactive metabolites like quinone imines are electrophilic; adducts are formed with different cellular components and molecules, i.e., proteins and lipids to function as neoantigen and to elicit B- and T-cell responses • Acyl glucuronides are toxic and may form adducts with proteins to result in immune cell-mediated injury	Boelsterli (2003), Kretz-Rommel and Boelsterli (1993), Lagas et al. (2010), Lee et al. (2016), Selvaraj et al. (2017), Tang (2003)
*Immune-mediated hepatitis in mice*			
KE1: Mitochondrial dysfunction in hepatocytes	High	• Diclofenac causes mitochondrial dysfunction and inhibits cellular respiration and ATP synthesis; histopathology reveals glycogen depletion to hallmark cellular stress • Repression of oxidative phosphorylation pathway as exemplified by Atp5a1, Atp6v0d2 and Ndufb6	Boelsterli (2003), Boelsterli and Lim (2007), Bort et al. (1999), Lee et al. (2016), Ramm and Mally (2013), Syed et al. (2016)
KE2: Induced apoptotic cell death in hepatocytes	High	• Increased expression of pro-apoptotic factors (caspase 8, FasL, interferon inducible death associated proteins like 1) • Bax/Bak-mediated mitochondrial outer membrane permeabilization and opening of the mitochondrial permeability transition pore (MPTP) in hepatocyte cultures results in ROS and cytochrome C release and programmed cell death	Gomez-Lechon et al. (2003a, b), Lagas et al. (2010), Lee et al. (2016), Masubuchi et al. (2002), Ramm et al. (2015), Sawa et al. (2009)
KE3: Induced ER stress/unfolded protein response in hepatocytes	High	• Reactive metabolites induce oxidative damage of ER components with induced expression of ER stress markers and inhibition of the proteasomal degradation of misfolded proteins	Lagas et al. (2010), Lee et al. (2016), Ramm et al. (2015)
KE4: Activation of immune cells	High	• Reactive metabolites and diclofenac adducts function as neo-antigens and stimulate immune cell responses of leukocytes, i.e., B-cell, T-cell, neutrophils, monocytes, Kupffer cells as well as APCs including hepatic stellate cells - Infiltration of immune cells in different regions of the liver - Increased expression of markers for activated macrophages and APCs (CD68, M-CSF, LBP, Ki67); inhibition of monocytes to differentiate into mature dendritic cells	Lee et al. (2016), Naisbitt et al. (2007), Sawa et al. (2009)
KE5: Activation of IFNγ signaling	High	• Genomic analysis revealed induced IFNγ signaling; immunohistochemistry confirms induction of fibronectin and M-CSF to hallmark tissue repair and differentiation of monocytes and macrophages. Transcript expression of the macrophage receptor with collagenous structure is strongly increased and immunohistochemistry of CD68 evidences activation of tissue-resident macrophages	Dutta et al. (2008), Lee et al. (2016), Yano et al. (2012)
KE6: Increased inflammation in hepatic sinusoidal space	High	• Diclofenac increases the expression of various chemokines and cytokines; inflammatory signaling pathways are activated - Increase of cytokines (IL1β and TNFα) in hepatocytes - Significant regulations of genes coding for cytokine receptors - Increased expression of proteins augmenting inflammation (CD44, S100a8, S100a9) - Remarkable modulation of inflammation by the adipocytokine receptor Lepr, the growth hormone receptor, protein tyrosine phosphatase non-receptor type 2 and sensors of cytokine signaling (Socs3)	Deng et al. (2009), Denson et al. (2001), Lee et al. (2016), Ramm and Mally (2013), Takayama et al. (1994)
AO: Immune-mediated hepatitis	High	• Immune-mediated hepatitis results in lobular inflammation and is hallmarked by inflammatory infiltrates, hepatic cholesterolosis, and phagocytosis • Serum biochemistry, histo- and immunohistopathology as well as genomic analysis demonstrate that diclofenac treatment causes an immune-mediated hepatitis	Lee et al. (2016)
*Immunoallergic hepatitis in dog*			
KE1: Mitochondrial dysfunction	Moderate	• Histopathology evidenced glycogen depletion to hallmark cellular stress and mitochondrial dysfunction	Selvaraj et al. (2017)
KE2: Induced apoptotic cell death	High	• Genomic analysis revealed apoptosis-related genes to highly regulated in diclofenac-treated dogs • Histopathology evidenced apoptotic cell death and apoptotic cellular degeneration in periportal and intermediated region (zone 1/2) of the liver	Selvaraj et al. (2017)
KE3: Induced microvesicular steatosis	High	• Significant changes in the expression of genes coding for lipogenesis, lipid transport, lipid droplet growth and fatty acid oxidation • Histopathology confirms microvesicular steatosis and vacuolated hepatocytes in the periportal and intermediate region	Selvaraj et al. (2017)
KE4: Mast cell activation	High	• Histopathology revealed mast cell activation and their infiltration into the sinusoidal space to evidence hypersensitivity/allergic reaction; marked mastocytosis - Strong induction of IgM, complement factors C4&B, SAA, SERPING1 - Marked induction of HIF1A and KLF6 in mast cells to hallmark oxidative stress and macrophage M2 polarization	Selvaraj et al. (2017)
KE5: Kupffer cell activation and polarization (M1/M2)	High	• The genomic and immunohistochemistry reveals activation and M2 polarization of Kupffer cells; migration of Kupffer cells into injured regions of the liver - Marked expression of CD205 and CD74 to facilitate antigen presentation and B-cell differentiation	Selvaraj et al. (2017)
KE6: Increased inflammation	High	• Induced expression of cytokines, chemokines and their receptors to regulate the trafficking of immune-competent cells to sites of inflammation • Pro-inflammatory cytokines and chemokines by macrophages and T/Th cells exacerbate liver injury (IFNr, IL-1, IL-6, IL17, Il18, CXCL1, CXCL2). Induced cytokines augment expression of acute-phase reactants like SAA and S100A8 - Increased VCAM-1 expression associated with leukocyte recruitment in vascular endothelium and sinusoidal regions marks inflammation - Increased MPO expression, a critical effector of inflammation in neutrophil, monocytes and macrophages	Selvaraj et al. (2017)
AO: Hypersensitivity/allergic hepatitis	High	• Diclofenac induced an immunoallergic hepatitis that is hallmarked by lobular inflammation, inflammatory cell infiltrates, hepatocellular damage and granulomatous hepatitis - Diclofenac treatment caused liver function test abnormalities with induced reticulocyte, WBC, platelet, neutrophil and eosinophil counts - Histopathology evidenced hepatic steatosis, acute lobular hepatitis, granulomas and mastocytosis	Selvaraj et al. (2017)

Evidence of essentiality was designated according to the OECD guidance as follows. High: direct evidence from specifically designed experimental studies illustrating prevention or impact on downstream KEs and/or the AO if upstream KEs are blocked or modified. Moderate: indirect evidence that modification of one or more upstream KEs is associated with a corresponding increase or decrease in the magnitude or frequency of downstream KEs. Low: no or contradictory experimental evidence of the essentiality of any of the KEs. Biological plausibility of KERs is suggested in supplementary Table S4

Diclofenac caused significant regulation of CYP monooxygenases after single and repeated treatment of mice (Lee et al. 2016). Independent investigations also support the key role of the acyl glucuronide produced by uridine diphosphoglucuronosyl transferase as a molecular initiation event (Oda et al. 2017; Seitz and Boelsterli 1998), and among NSAIDs the salicyl acyl glucuronide derived from aspirin is a further example. Thus, reactive metabolites are formed by hepatocytes, neutrophils and Kupffer cells with benzoquinone imine intermediates and acyl glucuronides being particularly harmful. If not sufficiently detoxified the reactive metabolites damage organelles, proteins and membrane lipids and eventually trigger programmed cell death. In specific, the accumulation of the reactive metabolite triggers oxidative stress and mitochondrial permeability transitions, i.e., mitochondrial toxicity by inhibiting ATP synthesis that leads to hepatocellular damage (Syed et al. 2016). Next to direct effects the reactive metabolites can covalently bind to proteins to form adducts. These function as neoantigens and are sensed and phagocytozed by APCs. Through interaction with the major histocompatibility complex APCs elicit B and T cell responses (Aithal 2011; Boelsterli 2003). In addition, diclofenac acyl glucuronide inhibits the *Mrp2* transport. This results in intrahepatic cholestasis and damage of the biliary epithelium (Boelsterli 2003; Lagas et al. 2010; Seitz and Boelsterli 1998). Similar to diclofenac the NSAIDs, lumiracoxib and indomethacin produce quinoneimine reactive intermediates, and next to ibuprofen and naproxen a wider range of carboxylic acid containing drugs are associated with allergic reactions (Stepan et al. 2011). The reactivity of acyl glucuronides derived from carboxylic acid containing drugs and the evidence for its toxicological concerns was recently summarized (Darnell et al. 2015; Van Vleet et al. 2017). Note, the covalent binding of acyl glucuronides to proteins constitutes a mechanism of toxicity, and the safety assessment of acyl glucuronides was the subject of a recent commentary with zomepirac being a prominent example for NSAID toxicity (Smith et al. 2018). Importantly, inhibition of MPO ameliorates adverse effects of MPO-derived oxidants (Malle et al. 2007) and MPO ko mice are an excellent system to study the importance of MPO in systemic inflammatory reactions. Alike, amelioration of diclofenac-induced toxicity was observed with cytochrome P450 reductase (CPR) null mice (Zhu and Zhang 2012), and multidrug resistance-associated protein 3 plays an important role in protection against acute toxicity of diclofenac acyl glucuronide as evidenced in *Mrp3*-null (KO) mice (Scialis et al. 2015). Moreover, NSAIDs are known to produce reactive oxygen species that result in cardiovascular disease (Ghosh et al. 2015), and a structural alert/reactive metabolite concept of 200 common drugs producing a wide range of different reactive metabolites was reported (Stepan et al. 2011). Collectively, the structural alerts quinoneimine and acyl glucuronides function as MIEs in immune-mediated and allergic hepatitis. Notwithstanding, defining an unequivocal MIE in the AOP framework can be complex as discussed in the seminal paper of Allen and coworkers (Allen et al. 2014).

#### KEs related to immune-mediated hepatitis

The biochemical and genomic data revealed diclofenac treatment to induce complex immune-mediated inflammatory signaling particularly from resident and migratory cells of the sinusoid and the space of Disse (Fig. 1 and Table 1). Within hepatocytes, the reactive metabolites elicit cellular stress responses including oxidative stress, mitochondrial dysfunction, apoptosis and ER stress/unfolded protein responses. In repeated-dose studies with mice, diclofenac treatment induced expression of the plasma membrane cysteine carrier (*Slc3a1*) to imply adaptive responses to oxidative stress. Furthermore, cysteine is an essential building block for the hepatic synthesis of reduced GSH and therefore of fundamental importance in alleviating oxidative stresses.

##### KE1: mitochondrial dysfunction

Diclofenac caused mitochondrial dysfunction through an inhibition of ATP synthesis (Boelsterli 2003; Kang et al. 2016; Syed et al. 2016). The significant repression of mitochondrial membrane transport proteins and key members of the oxidative phosphorylation pathway is testimony of an impaired mitochondrial respiration and ATP synthesis (Lee et al. 2016). Conversely, the plasma membrane Mg^2+^ transporter is strongly induced to increase intracellular Mg^2+^ concentration. Note increased Mg^2+^ uptake counteracts the detrimental effects of diclofenac treatment to alleviate mitochondrial stress and the opening of the Ca^2+^-dependent permeability transition pore to dampen apoptotic signaling.

##### KE2: apoptotic cell death

Reactive metabolites of diclofenac can directly or indirectly induce apoptotic cell death by activating several pro- and anti-apoptotic factors notably toll-like receptors, cytokine signaling inducible factors such as S100 calcium binding proteins and pro-inflammatory adipokines to augment ER stress-induced apoptosis (Lee et al. 2016; Sawa et al. 2009). Damaged hepatocytes send alarm signals like the damage-associated molecular patterns (DAMPs) that induce the immune and inflammatory response by activating immune cells. Although several DAMP molecules including S100 proteins were upregulated after repeated dosing of mice (supplementary Tables S2 and S3 for mice and dogs, respectively), the major components of the inflammasome are not regulated at the transcript level. Meanwhile, the danger hypothesis proposed that DAMPs can also be influenced by immune or inflammatory response. Our previous study demonstrated that diclofenac treatment induced the expression of inflammatory proteins which are released from macrophages/Kupffer cells. The subsequent inflammatory responses can reinforce the cellular damage of hepatocyte and in a vicious cycle strengthen inflammation (Lee et al. 2016).

##### KE3: ER stress/unfolded protein response

Genes involved in ER stress and unfolded protein response (UPR) were significantly regulated in the liver of diclofenac-treated mice. Independent studies evidenced diclofenac to trigger ER stress and UPR by PERK and ATF6 pathways as well as eIF2α phosphorylation (Foufelle and Fromenty 2016; Franceschelli et al. 2011; Fredriksson et al. 2014). However, the prolonged activation of PERK/eIF2α pathway induces apoptosis by activating the pro-apoptotic factor CHOP (Franceschelli et al. 2011; Fredriksson et al. 2014). In addition, ER stress can alter the lipid metabolism by UPR and leads to dyslipidemia (Basseri and Austin 2012). As reported by us, diclofenac caused hepatic cholesterolosis in mice with significant alteration in the transcription of genes coding for fatty acid and cholesterol metabolism (Lee et al. 2016). On the other hand, the repeated diclofenac treatment induced *Cyp7a1* expression which catalyzes the hydroxylation of cholesterol into bile acids (Lee et al. 2016). Alike, the induction of the apical sodium–bile acid transporter (*Slc10a2*) evidences changes in the transcellular transport of bile acids across the biliary epithelium to support the enterohepatic cycling of bile acids (Lee et al. 2016). Moreover, the organic anion transporter *Slc10a6* was induced and functions on taurolithocholic acid-3-sulfate (TCA-3S). Note, TCA-3S excretion into urine is about 90-fold higher in patients diagnosed with intrahepatic cholestasis of pregnancy thus highlighting its potential as a biomarker of hepatic cholestasis (Lee et al. 2016).

##### KE4: immune cell activation

Diclofenac adducts are sensed by APC and other phagocytic cells and trigger immune responses. The immune-mediated hepatitis is the result of complex interplay of innate and adaptive immune responses and involves the regulation of various cytokines/chemokines and their receptors (Lee et al. 2016). In specific, the released chemokines recruit the neutrophils, leukocytes and B lymphocytes to the sinusoidal space or to harmed hepatocytes, while cytokines endorse differentiation of myeloid and cytotoxic CD^8+^ T-cells (Saiman and Friedman 2012; Sawa et al. 2009). Increased expressions of interleukins modulate the activation and proliferation of T and/or NK cell responses (Hammerich and Tacke 2014; Zwirner and Domaica 2010) while members of the interleukin-1 superfamily stimulate the production of type 2 cytokines by T-helper cells (Miller 2011). Thus, diclofenac treatment resulted in an activation of several cytokines to affect T cell differentiation. Collectively, diclofenac stimulated an activation of diverse immune cells including monocytes, Kupffer cells and APC (Lee et al. 2016).

##### KE5: IFNγ signaling

Increased expression of IFNγ hallmarks innate and adaptive immune responses. IFNγ plays a pivotal role in host defense in response to infections and mediating the inflammation by producing the pro-inflammatory cytokines (Muhl and Pfeilschifter 2003). Genomic analysis revealed protein tyrosine phosphatase non-receptor type 2 (*Ptpn2*) to be significantly regulated in mice after diclofenac treatment and *Ptpn2* plays a critical role in modulating IFNγ signaling (Lee et al. 2016; Scharl et al. 2010). Together, diclofenac treatment induced expression of inflammatory cytokines including *IFNγ*, interleukins and *TNFα* (Dutta et al. 2008; Yano et al. 2012).

##### KE6: inflammation

Our computational studies defined key master regulatory molecules and their associated networks. Based on independent RT-qPCR studies, induced expression of suppressor of cytokine signaling (*Socs*), leptin, growth hormone receptor (*Ghr*), and Ptpn proteins was confirmed and these function in IFNγ, Jak/Stat, pro- and anti-inflammatory signaling pathways. *Stat3* is one of the major transcription factors activated by cytokines and growth factors to influence pro-inflammatory (*Mapk*, *p38*, *Jnk*, and IκB kinase) and anti-inflammatory signaling (Pi3k-Akt) events. Among individual animals marked induction of this protein was observed which controls transcription of Socs and inhibits Jak/Stat3 signaling (Kong et al. 2002). In addition, leptin and other adipokine signaling molecules play a major role in energy intake to influence monocyte and macrophage activity during inflammation (Fantuzzi and Faggioni 2000). Next to its role in the control of energy intake leptin and its receptor modulates Jak/Stat, Erk 1/2 and Pi3k signaling (Bjorbaek and Kahn 2004; Cottrell and Mercer 2012; Paz-Filho et al. 2012) and therefore plays a decisive role in inflammation. Similarly, the growth hormone receptor is influenced by multiple intracellular signaling cascades (Jak–Stat and chemokine signaling) and functions in liver regeneration. Diclofenac treatment reinforced *Ghr* degradation as evidenced by immunoblotting to suppress hepatic *Ghr* signaling (Takahashi 2017). Diclofenac also induced expression of the endothelial–leukocyte adhesion molecule selectin which recruits leukocytes to the inflammatory site and the computational analysis defined selectin as a master regulator (Lee et al. 2016; Ley 2003). Furthermore, the highly significant induction of lipocalin-2 (*Lcn2*) signifies sterile inflammation and neutralization of LCN2 controls neutrophilic inflammation as had been summarized (Moschen et al. 2017). Taken together, the IFNγ, Jak/Stat, adipocytokine and chemokine signaling pathways provide a rationale for the AOP of immune-mediated hepatitis observed in mice in response to diclofenac treatment.

### AOP for diclofenac-induced immunoallergic hepatitis in dogs

**Fig. 2:**
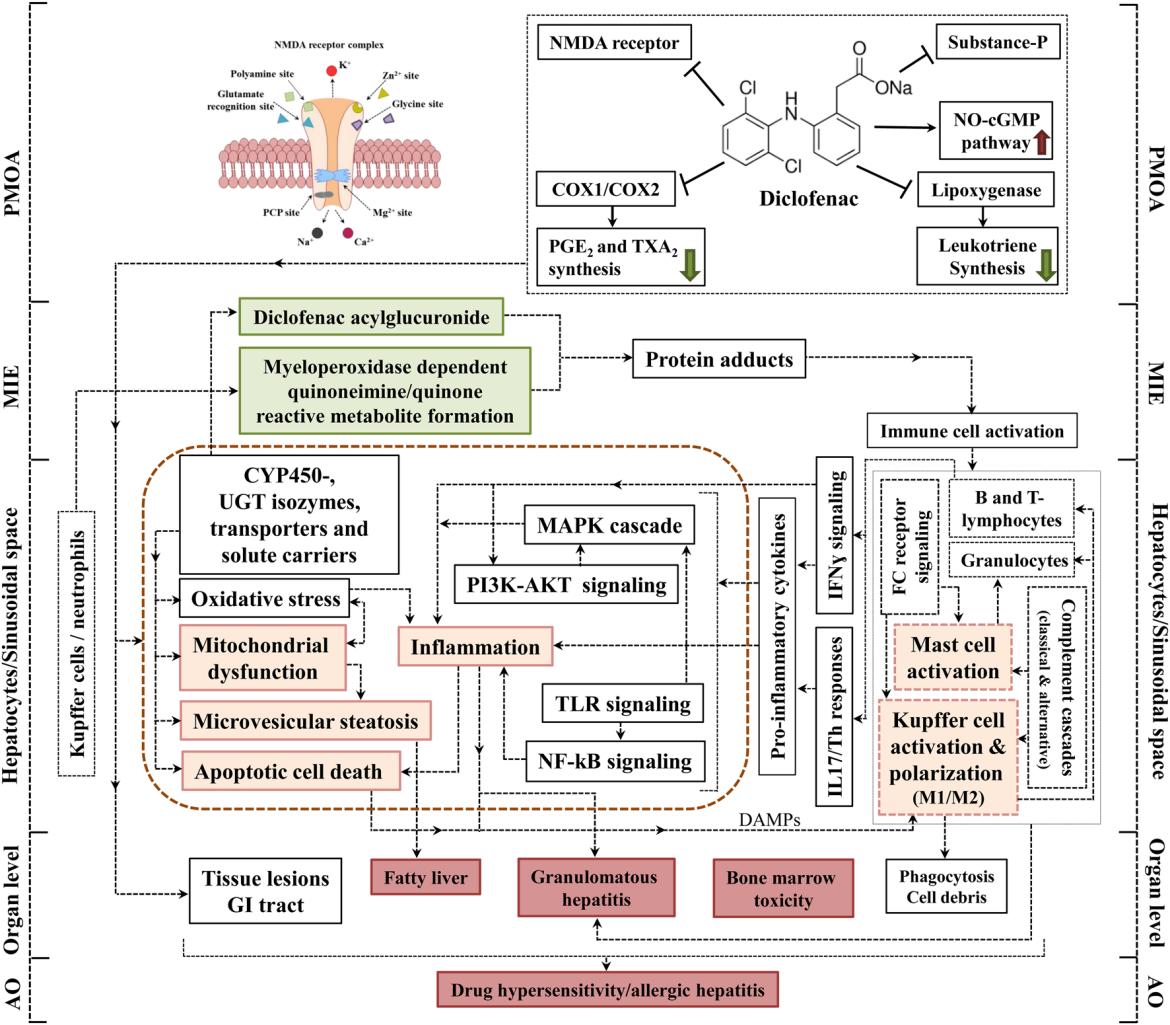
AOP of immunoallergic hepatitis in dog in response to diclofenac. Diclofenac induced the key molecules related to the liver hypersensitivity reactions including oxidative stress, macrophage polarization, mastocytosis, complement activation and an erroneous programming of the innate and adaptive immune system that can cause granulomatous hepatitis. Putative MIE and KEs associated with immunoallergic hepatitis in dogs were highlighted; green boxes represent MIE, orange color is for KEs in the cellular level, and red-colored boxes define the KEs in organ and organism level. The potential linkages among the KEs are shown based on the experimental evidences and literature. The brown dotted line demonstrates the KEs occurred in the hepatocytes

To the best of our knowledge an animal model to investigate allergic hepatitis has not been developed so far. Our studies highlight the relevance of dogs as a surrogate for clinical immunoallergic DILI and its effects on the immune system. Further justification of the canine model resides in the similar expression of COX1/COX2 between dogs and humans (Kay-Mugford et al. 2000; Radi and Khan 2006; Radi 2009). To investigate diclofenac’s mechanism of immuno-allergic hepatitis, beagle dogs were given 1 or 3 mg/kg/day for 28 days. Histopathology revealed micro- and macrovesicular hepatic steatosis, glycogen depletion, apoptosis, acute lobular hepatitis, granulomas and mastocytosis. Biochemical and whole genome scans revealed that diclofenac induced hypersensitivity reactions. Key molecules related to oxidative stress, macrophage polarization, mast cell activation and complement cascade were regulated as the result of an erroneous programming of the innate and adaptive immune system to cause granulomatous hepatitis (Selvaraj et al. 2017). The AOP of immune-allergic hepatitis is depicted in Fig. 2 and Table 1.

#### MIE

With dogs the MIE is similar to the one described for mice, i.e., the formation of iminoquinone and acyl glucuronide reactive metabolites. However, the abundance of metabolite formation differs among species (Fujiwara et al. 2018; Hughes and Swamidass 2017; Pulli et al. 2013; Sasaki and Yokoi 2018; Smith et al. 2018).

#### KEs related to immunoallergic hepatitis

The genomic study revealed significant changes in the regulation of genes coding for immune, inflammation, apoptosis and oxidative stress responses to diclofenac treatment. In specific, the reduced expression of hepatic CYP monooxygenases and phase II enzymes is caused by inflammation and the immune response. Induced expression of acute-phase proteins (SAA), alpha-macroglobulin, fibrinogen, complement factors and cytokines evidences sterile inflammation. The significant up-regulation of mainly positive acute-phase proteins evidences inflammation in response to reactive metabolites and harmed hepatocytes; notwithstanding, hepatic SOD1 protein expression is decreased after diclofenac treatment as a result of oxidative stress.

##### KE1/KE2: mitochondrial dysfunction and apoptotic cell death

In addition, reactive metabolites of diclofenac cause cellular stress and increased the level of ROS which leads to mitochondrial damage and subsequent apoptosis in hepatocytes (Boelsterli 2003; Gomez-Lechon et al. 2003a). Consistent with the findings observed with mice, the genomic analysis of dog liver discovered genes related to oxidative stress, mitochondrial biogenesis and membrane transport and apoptosis as significantly regulated (Selvaraj et al. 2017).

##### KE3: microvesicular steatosis

Oxidative stress and impaired mitochondrial activity can result in drug-induced steatosis (Pessayre 2007; Sahini et al. 2014). The genes coding for lipogenesis, lipid transport, lipid droplet growth, ER stress and fatty acid oxidation were significantly regulated (Selvaraj et al. 2017) and histopathology evidenced hepatic steatosis in diclofenac-treated dogs. Note, drugs other than NSAIDs were reported to cause drug-induced steatosis in animal models and patients (Freneaux et al. 1990; Patel and Sanyal 2013; Sahini et al. 2014).

##### KE4: mast cell activation

Diclofenac caused mast cell activation and hepatic infiltration with strong induction of immunoglobulins, Fc-receptor signaling molecules and acute-phase proteins as well as the classical and alternative pathway components of the complement system to highlight allergic reactions. The marked mastocytosis hallmarks drug hypersensitivity. It is associated with granulocyte and mast cell degranulation and the release of pro-inflammatory mediators including histamines, prostaglandins, leukotrienes and other cytotoxic molecules (Selvaraj et al. 2017; Theoharides et al. 2012; Zhang et al. 2018) to aggravate the inflammatory responses and support migration of other inflammatory immune cells to sites of injury. Thus, the AOP highlights activation of the complement system as a mechanism of toxicity resulting in granulomatous hepatitis.

##### KE5: Kupffer cell activation and/polarization (M1/M2)

Diclofenac treatment caused an activation of Kupffer cells and strong expression of M1/M2 marker genes (Selvaraj et al. 2017). Activated Kupffer cells release a range of inflammatory mediators, growth factors and acute-phase proteins to perpetuate liver inflammation (Kolios et al. 2006; Roberts et al. 2007). Moreover, through complex cellular cross-talks macrophages influence the differentiation of Th-cell populations including Th17. Therefore, the induction of markers of M2-polarized Kupffer cells like IL10, IL4/IL13, the innate immune response (CD14), the scavenger receptors (CD163, MARCO and CXCL16), MHC class II molecules (CD74 and HLA-DRB1) as well as their sensor and effectors (complement genes C1QA-C, IGF) are suggestive for Th2 responses to alleviate the inflammatory reactions to diclofenac treatments (Selvaraj et al. 2017). Moreover, histopathology evidenced inflammatory infiltrates like immature/migrating macrophages, Kupffer cells, granulocytes and lymphocytes into interstitial and the sinusoidal space as well as harmed hepatic parenchyma (Selvaraj et al. 2017).

##### KE6: inflammation

Diclofenac treatment caused complex pro- and anti-inflammatory reactions in the liver (Chen et al. 2015; Njoku 2014; Yano et al. 2012). In specific, the pro-inflammatory chemokines and their receptors play a major role in cytotoxic T-cell activation and the trafficking of inflammatory immune cells (neutrophils, leukocytes, B-lymphocytes) to sites of injury (Karin 2010; Wong and Fish 2003). In addition, MAPK signaling molecules were induced in response to diclofenac treatment and their regulation can be triggered by cellular stress and pro-inflammatory cytokines to result in inflammation-mediated hepatotoxicity (Guegan et al. 2013; Kyriakis and Avruch 2012; Nakagawa and Maeda 2012; Nikolaou et al. 2013). Importantly, increased expression of TNF family members activates NF-κB, JNK, p38, and ERK1/ERK2 signaling and are involved in T-cell and T-helper (Th1, Th2 and Th17) cell-mediated responses and hepatic inflammation (Aiba and Nakamura 2013; Akiyama et al. 2012; Sakai et al. 2012; Zhang and Li 2012), whereas significant repression of the LY6 antigen indicates adaptive response to cytokine-induced inflammation (Begue et al. 2006). Collectively, the cytokines and chemokines bind and activate their corresponding receptors to promote immune-mediated inflammation of the liver.

**Fig. 3:**
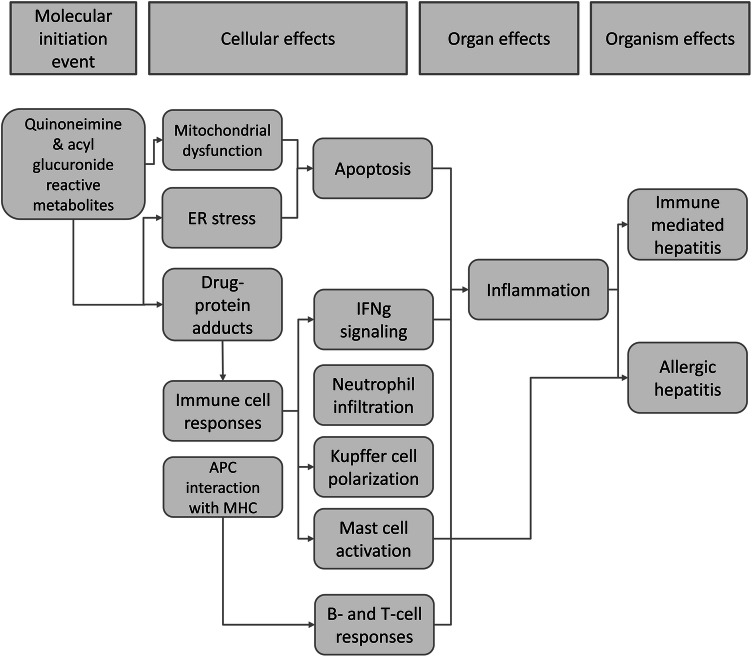
A simplified AOP for immune-mediated and allergic hepatitis

The biological plausibility of KERs is summarized in supplementary Table S4 and Fig. 3 provides a simplified AOP for diclofenac-induced immune-mediated and allergic hepatitis.

### Clinical relevance

A long-term prospective clinical trial involving 17,289 arthritis patients who were randomly assigned to diclofenac (150 mg daily) or etoricoxib (60 or 90 mg daily) evidenced diclofenac to cause common aminotransferase elevations (Laine et al. 2009). Alike, a systematic review of randomized clinical trial data of 3 NSAIDs revealed diclofenac to be top ranking for hepatotoxic events (Sriuttha et al. 2018). Earlier studies already reported cases of acute hepatitis induced by diclofenac (Helfgott et al. 1990; Iveson et al. 1990; Purcell et al. 1991; Sallie 1990), and liver biopsy findings defined a histological injury pattern of granulomatous hepatitis. Note, our studies with dogs also demonstrated granulomatous hepatitis as a key finding (see above KE4) and the granulomas are composed of inflammatory cells and histocytes (Ramachandran and Kakar 2009). Moreover, a current study compared 30 DILI cases among 8 NSAIDs and found diclofenac to be the most frequently implicated NSAID. The cases are characterized by hepatocellular injury, prolonged hospitalization and included a patient with fatal Stevens–Johnson syndrome (Schmeltzer et al. 2015). In their study 38% of diclofenac DILI cases presented fever, rash and eosinophilia, and the findings are consistent with the clinical features of drug hypersensitivity reactions (Schmeltzer et al. 2015). Altogether, the proposed AOP of immune-mediated and allergic hepatitis is relevant for human DILI induced by NSAIDs.

### How can the AOP concept be translated into clinical and regulatory practice?

Biomarkers based on AOPs carry the potential to significantly improve an assessment of adverse drug reactions (ADRs). In specific, ADRs are assessed by expert opinion and the guidance given by the WHO and regulatory authorities. Furthermore, the magnitude and the incidence of ADRs are evaluated by postmarketing surveillance and the legally required period safety updated reports (PSUR). Although ADRs on single-drug treatment can be evaluated with certainty, the causality assessment of ADRs among comorbid patients which typically involves several drugs can be confounded by the complex drug properties–host factor interactions that need to be deciphered. Adding to complexity is the fact that scoring systems to evaluate organ specific toxicities are spares and with the exception of drug-induced liver injury, i.e., the RUCAM (Roussel Uclaf Causality Assessment Method) and the MELD (Model for End Stage Liver Disease) score in liver transplantation, there are no algorithms to assess more objectively organ-specific ADRs.

By probing for mechanistically plausible key events associated with drug injury, AOPs can help to define biomarkers to improve the causality assessment of ADRs. The development of AOPs for different drug classes and the clinical validation of AOP-defined biochemical markers will be a priority task.

## Conclusion

Diclofenac treatment induced divergent immune responses among two important animal species commonly used in toxicity studies. The knowledge gain from these studies will be the base for the development of an integrated AOP for immune-mediated hepatitis.

## Electronic supplementary material

Below is the link to the electronic supplementary material.

Supplementary file1 (DOCX 23 kb)

Supplementary file2 (XLS 34 kb)

Supplementary file3 (XLS 35 kb)

Supplementary file4 (DOCX 21 kb)

## Abbreviations

ADR: Adverse drug reaction
ALT: Alanine aminotransferase
AOP: Adverse outcome pathways
APC: Antigen-presenting cells
AST: Aspartate aminotransferase
ATF6: Activating transcription factor 6
Atp5a1: ATP synthase F1 subunit alpha
Atp6v0d2: ATPase H + transporting V0 subunit D2
C1QA-C: Complement C1q A chain
CD14/68/74/163/205: Cluster of differentiation 14/68/74/163/205
CHOP: C/EBP homologous protein
COX 1/2: Cyclooxygenase 1/2
CXCL16: C-X-C motif chemokine ligand 16
CYP: Cytochrome P450
Cyp7a1: Cytochrome P450 family 7 subfamily A member 1
DAMPs: Damage-associated molecular patterns
DILI: Drug-induced liver injury
eIF2α: Eukaryotic translation initiation factor 2A
ER: Endoplasmic reticulum
Erk: Extracellular signal-regulated kinase
FasL: Fas ligand
Ghr: Growth hormone receptor
GSH: Glutathione
HIF1A: Hypoxia inducible factor 1 subunit alpha
HLA-DRB1: Major histocompatibility complex, class II, DR beta 1
IFNγ: Interferon gamma
IGF: Insulin like growth factor 1
IL4/10/13: Interleukin 4/10/13
Jak: Janus kinase
Jnk: C-Jun N-terminal kinase
KE: Key event
KER: Key event relationship
KLF6: Kruppel like factor 6
LBP: Lipopolysaccharide binding protein
LCN2: Lipocalin 2
LY6: Lymphocyte antigen-6
Mapk: Mitogen-activated protein kinase 1
MARCO: Macrophage receptor with collagenous structure
M-CSF: Macrophage colony-stimulating factor
MELD: Model for end-stage liver disease
MHC: Major histocompatibility complex
MIE: Molecular initiating event
MPO: Myeloperoxidase
MPTP: Mitochondrial permeability transition pore
Mrp2: Multidrug resistance-associated protein 2
Ndufb6: NADH:Ubiquinone oxidoreductase subunit B6
NGS: Next-generation sequencing
NK: Natural killer
NMDA: *N*-Methyl-d-aspartate receptor
NO: Nitric oxide
NSAID: Non-steroidal anti-inflammatory drug
PERK: Protein kinase RNA-like ER kinase
PI3K: Phosphoinositide 3-kinases
PPARɣ: Peroxisome proliferator-activated receptor gamma
Ptpn2: Protein tyrosine phosphatase non-receptor type 2
PSUR: Periodic safety update report
ROS: Reactive oxygen species
RUCAM: Roussel Uclaf Causality Assessment Method
S100a8/9: S100 Calcium binding protein A8/9
SAA: Serum amyloid A
SERPING1: Serpin family G member 1
Slc10a2/6: Solute carrier family 10 member 2/6
Slc3a1: Solute carrier family 3 member 1
Socs: Suppressor of cytokine signaling
SOD1: Superoxide dismutase 1
Stat3: Signal transducer and activator of transcription 3
TCA-3S: Taurolithocholic acid-3-sulfate
TNFα: Tumor necrosis factor alpha
UGT2B7: UDP glucuronosyltransferase family 2 member B7
UPR: Unfolded protein response
VCAM-1: Vascular cell adhesion molecule 1
WBC: White blood cells
