# Supplementary material for: An adverse outcome pathway for immune-mediated and allergic hepatitis: a case study with the NSAID diclofenac
Source: Arch Toxicol. 2020 May 5;94(8):2733–48. doi: 10.1007/s00204-020-02767-6 (PMC7395045; doi:10.1007/s00204-020-02767-6)
Supplement: Supplementary file 1 — Supplementary file1 (DOCX 23 kb) [file 204_2020_2767_MOESM1_ESM.docx]

**Supplementary Table 1**: Drugs/Chemicals metabolized to quinone imine reactive intermediates

| **Chemical/drug** | **Category** | **Reactive intermediates** | **Biological Effect/toxicity** | **Reference** |
| --- | --- | --- | --- | --- |
| Carvedilol | Alpha and beta-blockers | Catechol and *ortho*-quinone | Mixed-pattern of hepatitis (hepatocellular inflammation and necrosis and mild signs of chronic cholestasis) | PMID:31192288, PMID: 11724083 |
| Lapatinib | Anti-cancer | Quinone imine | Drug induced hepatitis, hepatic inflammation (inflammation with infiltration of neutrophil and eosinophil in portal area, focal necrosis in lobular area, apoptotic body in periportal area) | PMID:21633602, PMID: 26036634, PMID: 22100178 |
| Carbamazepine | Anticonvulsants | Quinone imine and *ortho*-quinone | Cholestatic hepatitis, idiosyncratic liver injury (inflammasome activation), hepatic necrosis with inflammation, granulomatous hepatitis, hypersensitivity-associated liver injury (eosinophilia) | PMID: 29200572, PMID: 31677073, PMID: 7282758, PMID: 22790970 |
| Nefazodone | Antidepressant | Quinone imine and benzoquinone | Hepatitis and hepatocellular necrosis. Inactivation of P450. Mitochondrial toxicant and inhibition of bile transporter | PMID: 16410371, PMID: 12212533, PMID: 12025437 |
| Trazodone | Antidepressant | Quinone imine and epoxide | Acute hepatitis (mixed or choestatic), immunoallergic (eosinophilia), hepatic necrosis, intrahepatic cholestasis, modest inflammation | PMID: 10685763, PMID: 11793619, PMID: 28848778 |
| Tamoxifen | Antiestrogens | O-quinine | Fatty liver and steatohepatitis | PMID: 8610959 , PMID: 31479913, PMID: 26071793, PMID: 27240168 |
| Amodiaquine | Antimalarial | Quinone imine | Immune mediated DILI (Kupffer cell activation, apoptosis, hepatocyte proliferation and activated M2 macrophages) | PMID: 27416278, PMID: 25046026, PMID: 26154582 |
| Aripiprazole | Antipsychotic | Quinone imine | Drug induced hepatitis ( portal inflammatory cellular reaction with eosinophilia) | DOI: 10.4172/2329-6887.1000201, PMID: 31671495 |
| Quetiapine | Antipsychotic drug | quinone imine | Cholestatic hepatitis | PMID: 19047843 |
| Chlorpromazine | Antipsychotic drug | quinone imine | Intrahepatic cholestasis, liver and bone marrow granulomas associated with agranulocytosis | PMID: 2401609 |
| Paracetamol | Antipyretic and analgesic | Quinone imine | Sterile inflammation, mitochodrial dysfunction, immune mediated DILI | PMID: 29753208, PMID: 21745276 |
| Phenytoin | Anti-seizure | O-quinine | Cholestatic hepatitis, acute immunoallergic hepatitis (fever, rash, eosinophilia) | PMID: 6721716, PMID: 30155393 |
| Tolcapone | COMT inhibitor | O-quinine or quinone imine | Hepatotoxicity (mitochondrial dysfunction and bile acid transport inhibition) | PMID: 26844013 |
| Thalidomide | Immunomodulatory agent | O-quinine | Acute cholestatic hepatitis, hepatocellular necrosis and inflammatory cell infiltration | PMID: 22789729 |
| Desatinib | Kinase inhibitor  (blood cancer) | Quinone imine, imine-methide | Hepatotoxicity (inactivation of CYP450, oxidative stress mediated apoptosis) | PMID: 19282395, PMID: 22538170 |
| Erlotinib | Kinase inhibitor (EGFR inhibitor) (non-small cell lung cancer and  pancreatic cancer) | Quinone imine | Hepatotoxicity (Inactivation of P450, hyperbilirubinemia, mitochondrial pathway‑mediated apoptosis) | PMID: 29387241, PMID: 17575239 , PMID: 17228132 |
| Gefitinib | Kinase inhibitor (EGFR inhibitor) (non-small cell lung cancer) | Quinone imine | Chronic hepatitis with active necrosis | PMID: 16293881 |
| Lumiracoxib | NSAID | Benzoquinone imine | Hepatotoxicity (severe hepatic necrosis, oxidative stress, altered immune response) | PMID: 22142375 |
| Indomethacin | NSAID | quinone imine | Hepatotoxicity (Oxidative stress, micro/macro vesicular steatosis) | PMID: 16771604, PMID: 31080746 |
| Raloxifene | Selective estrogen receptor modulators | O-quinone | Cholestatic hepatitis, hepatic steatosis and lobular inflammation | PMID: 9820309, PMID: 23201442, PMID: 25868633 |

Selvaraj S, Oh JH, Borlak J. An adverse outcome pathway for diclofenac induced immune mediated and allergic hepatitis. Archives of Toxicology

Corresponding author: Prof. Dr. Jürgen Borlak, Hannover Medical School, Centre for Pharmacology and Toxicology, 30625 Hannover, Germany,

E-mail: borlak.juergen@mh-hannover.de
