# Supplementary material for: An adverse outcome pathway for immune-mediated and allergic hepatitis: a case study with the NSAID diclofenac
Source: Arch Toxicol. 2020 May 5;94(8):2733–48. doi: 10.1007/s00204-020-02767-6 (PMC7395045; doi:10.1007/s00204-020-02767-6)
Supplement: Supplementary file 4 — Supplementary file4 (DOCX 21 kb) [file 204_2020_2767_MOESM4_ESM.docx]

**Supplementary Table 4. Support for biological plausibility of KERs**

| Support for biological plausibility of KERs | Defining Question | High | Moderate | Low |
| --- | --- | --- | --- | --- |
|  | Is there a mechanistic (i.e., structural or functional) relationship between KE_up_ and KE_down_ consistent with established biological knowledge? | Extensive understanding based on extensive previous documentation and broad acceptance. | The KER is plausible based on analogy to the accepted biological relationships but scientific understanding is not completely established. | There is empirical support for a statistical association between KEs, but the structural or functional relationship between them is not understood. |
| *Immune-mediated hepatitis* | | | | |
| MIE => KE1: Reactive metabolites leading to mitochondrial dysfunction | High:  There is extensive evidence that reactive metabolite quinone imine leads to mitochondrial dysfunction. Mitochondria is an important target of the reactive metabolite and they cause the mitochondrial dysfunction by protein adduct or altered redox balance. | | | |
| KE1 => KE2: Mitochondrial dysfunction leading to apoptosis | High:  There is extensive evidence that mitochondrial dysfunction leads to apoptosis. Mitochondrial dysfunction with activation of the mitochondrial permeability transition (MPT) pore results in the release of pro-apoptotic factors leading to apoptotic cell death. | | | |
| KE1 => KE3: Mitochondrial dysfunction leading to ER stress/unfolded protein response | High:  There is extensive evidence that increased mitochondrial dysfunction result in reactive oxidative stress (ROS) production and then contributes to activation of ER stress/unfolded protein response. | | | |
| MIE => KE4: Reactive metabolite leading to immune cell activation | High:  There is extensive evidence that quinone imine and acylglucuronide metabolites lead to immune cell activation through their covalent binding to biomolecules. Quinones and acylglucuronide metabolites can readily react with cysteine in proteins and these protein adducts act as neoantigens for immune cell activation. | | | |
| KE4 => KE5: Immune cell activation leading to IFNɣ signaling | High:  There is extensive evidence that IFNɣ is produced predominantly by immunce cells including natural killer (NK) and T cells. | | | |
| KE5 => KE6: IFNɣ signaling leading to inflammation | High:  It is broadly accepted that increased expression of IFNγ hallmarks innate and adaptive immune responses. IFNγ plays a pivotal role in host defence in response to infections and mediating the inflammation by producing the pro-inflammatory cytokines | | | |
| KE2 => KE6: Apoptosis leading to inflammation | High:  There is extensive evidence that apoptotic cell death plays an important role in the result of inflammation. Damage associated molecular patterns (DAMPs) are the key to trigger the inflammatory reaction | | | |
| *Immunoallergic hepatitis* | | | | |
| MIE => KE1: Reactive metabolites leading to mitochondrial dysfunction | High: (same above)  There is extensive evidence that reactive metabolite including quinone imine leads to mitochondrial dysfunction. Mitochondria is an important target of the reactive metabolite and they cause the mitochondrial dysfunction by protein adduct or altered redox balance. | | | |
| KE1 => KE2: Mitochondrial dysfunction leading to apoptosis | High: (same above)  There is extensive evidence that mitochondrial dysfunction leads to apoptosis. Mitochondrial dysfunction with activation of the mitochondrial permeability transition (MPT) pore results in the release of pro-apoptotic factors leading to apoptotic cell death. | | | |
| KE1 => KE3: Mitochondrial dysfunction leading to microvesicular steatosis | Moderate:  It is broadly accepted that microvesicular steatosis presents as accumulation of small droplets in the hepatocytes and it is usually associated with mitochondrial dysfunction. | | | |
| MIE => KE4: Reactive metabolite leading to mast cell activation | High:  It is broadly accepted that a number of factors including antigen, IgE or hormone can trigger mast cell activation. The reactive metabolites results in covalent binding to biomolecules which act as neoantigen. The neoantigens trigger immune cell activation and subsequent mast cell activation. | | | |
| MIE => KE5: Reactive metabolite leading to Kupffer cell activation & polarization (M1/M2) | Moderate:  There is extensive evidence that the main activators of Kupffer cells are immune molecules and antigen like LPS. The reactive metabolite activates the immune response like complement factors and lead to Kupffer cell activation. | | | |
| KE4 => KE6: Mast cell activation leading to inflammation | High:  There is extensive evidence that mast cells play key roles in regulation of inflammation and immune response. Mast cell can recruit the immune cells including T cells at peripheral site of inflammation by secreting cytokines. | | | |
| KE5 => KE6: Kupffer cell activation leading to inflammation | High:  It is broadly accepted that the M1 and M2 polarization involve in immune response and tissue repair. Activated Kupffer cells release wide range of inflammatory mediators, growth factors, acute phase proteins to perpetuate liver inflammation. | | | |

Selvaraj S, Oh JH, Borlak J. An adverse outcome pathway for diclofenac induced immune mediated and allergic hepatitis. Archives of Toxicology

Corresponding author: Prof. Dr. Jürgen Borlak, Hannover Medical School, Centre for Pharmacology and Toxicology, 30625 Hannover, Germany,

E-mail: borlak.juergen@mh-hannover.de
